# Impact of genetic variants in clinical outcome of a cohort of patients with oropharyngeal squamous cell carcinoma

**DOI:** 10.1038/s41598-020-66741-z

**Published:** 2020-06-19

**Authors:** Ana Carolina de Carvalho, Sandra Perdomo, Wellington dos Santos, Gabriela Carvalho Fernandes, Lais Machado de Jesus, Raiany Santos Carvalho, Cristovam Scapulatempo-Neto, Gisele Caravina de Almeida, Bruna Pereira Sorroche, Lidia Maria Rebolho Batista Arantes, Matias Eliseo Melendez, Pedro De Marchi, Neil Hayes, Rui Manuel Reis, André Lopes Carvalho

**Affiliations:** 1 Molecular Oncology Research Center, Barretos Cancer Hospital, Barretos, SP Brazil; 2 Institute of Nutrition, Genetics and Metabolism Research, Faculty of Medicine, Universidad El Bosque, Bogotá, Colombia; 3 International Agency of Research on Cancer, Lyon, France; 4 Center of Molecular Diagnosis, Barretos Cancer Hospital, Barretos, SP Brazil; 5 Research Support Center, Barretos Cancer Hospital, Barretos, SP Brazil; 6 Pathology and Molecular Diagnostics Service, Diagnósticos da América-DASA, São Paulo, SP Brazil; 7 Pelé Little Prince Research Institute, Curitiba, PR Brazil; 8 Little Prince College, Curitiba, PR Brazil; 9 Department of Medical Oncology, Barretos Cancer Hospital, Barretos, SP Brazil; 10 Oncoclinicas, Rio de Janeiro, RJ Brazil; 11 Department of Medicine, Division of Oncology, UTHSC Center for Cancer Research, University of Tennessee Health Science Center, Memphis, USA; 12 Life and Health Sciences Research Institute (ICVS), Medical School, University of Minho, Braga, Portugal; 13 ICVS/3B’s-PT Government Associate Laboratory, Braga/Guimarães, Portugal

**Keywords:** Cancer, Cancer epidemiology, Cancer genetics, Cancer genomics, Head and neck cancer, Oral cancer

## Abstract

Tobacco- or human papillomavirus- driven oropharyngeal squamous cell carcinomas (OpSCC) represent distinct clinical, biological and epidemiological entities. The aim of this study was to identify genetic variants based on somatic alterations in OpSCC samples from an admixed population, and to test for association with clinical features. The entire coding region of 15 OpSCC driver genes was sequenced by next-generation sequencing in 51 OpSCC FFPE samples. Thirty-five percent of the patients (18/51) were HPV-positive and current or past tobacco consumption was reported in 86.3% (44/51). The mutation profile identified an average of 2.67 variants per sample. Sixty-three percent of patients (32/51; 62.7%) were mutated for at least one of the genes tested and *TP53* was the most frequently mutated gene. The presence of mutation in *NOTCH1* and *PTEN*, significantly decreased patient’s recurrence-free survival, but only *NOTCH1* mutation remained significant after stepwise selection, with a risk of recurrence of 4.5 (HR 95% CI = 1.11–14.57; Cox Regression p = 0.034). These results show that Brazilian OpSCC patients exhibit a similar clinical and genetic profile in comparison to other populations. Molecular characterization is a promising tool for the definition of clinical subgroups, aiding in a more precise tailoring of treatment and prognostication.

## Introduction

Head and neck cancers (HNC) are ranked as the 8^th^ most common tumors worldwide, with a total of 834,860 new cases and 431,131 deaths estimated for 2018^1^.

The most prevalent site is the oral cavity, followed by the oropharynx and larynx, and more than 95% are squamous cell carcinomas (SCC)^2^. Excessive tobacco and alcohol consumption and high-risk human papillomavirus (HPV) infection (mainly HPV-16 and HPV-18) in the oropharynx are the main risk factors^3–6^. Besides etiology, HPV-positive and HPV-negative oropharyngeal squamous cell carcinoma (OpSCC) cases configure diseases with remarkably different clinical presentation, epidemiological and molecular profiles^3–6^. Regarding prognosis, HPV-positive OpSCC have a far more favorable outcome when compared with HPV-negative cases^6^. For this reason, the tumor-node-metastasis (TNM) staging for HNC was adapted in the eighth edition to include p16^INK4A^ immunostaining as a surrogate for HPV status in OpSCC^7^.

Treatment for OpSCC has evolved in the past decades and often includes approaches for organ preservation based on concomitant chemotherapy and radiotherapy followed by salvage surgery in non-responders^8^. This treatment frequently has long-term side effects, impacting patients’ quality of life^9^. Several treatment de-escalation trials for HPV-positive OpSCC have been initiated, which may lead to personalized treatment based on HPV status^10^.

Around 2/3 of HNC patients are diagnosed with advanced disease in different parts of the world^9,11–13^ and at least 50% of them will relapse locally, regionally or at distant sites, within the first two years after treatment^9,14,15^. While for HPV-positive OpSCC cases, 5-years overall survival rates (OS) are close to 80%, regardless of stage^16–18^, TNM stage has a significant impact in OS for HPV-negative OpSCC: 70%, 58%, 50%, and 30% for stage I, II, III, and IV^18^. Therefore, there is an increasing need for predictors of treatment response and disease progression/relapse.

Studies characterizing the spectrum of genetic alterations in cancers have been enabling a better understanding of the molecular alterations playing a role in head and neck carcinogenesis^19–22^. A high level of intertumoral heterogeneity has been observed, confirming the biological complexity of these tumors^22–24^. A common finding is that HPV-negative tumors have a higher mutation burden while HPV-positive tumors have far fewer genes mutated and variants per tumor, regardless of smoking status^22,23^. Results from The Cancer Genome Atlas (TCGA) consortium on the molecular profile of 279 HNSCC tumors confirmed the differences at the molecular level between HPV-positive and negative cases, with frequent activating helical domain mutations in *PIK3CA* in HPV-positive cases and *TP53* mutations and *CDKN2A* loss-of-function mutations in HPV negative cases^24^. These findings suggest that the absence of the oncogenic effect from HPV oncoproteins in HPV-unrelated tumors requires the accumulation of multiple genetic aberrations to allow malignant transformation^23^.

Despite the different strategies for diagnosis and treatment of OpSCC patients, and the advent of HPV as an important prognostic marker, there is no molecular biomarker to guide selection among treatment and follow-up options that directly have an impact on patient survival. Therefore, the lack of risk categories based on clinical features and/or biomarkers that can be used for personalized treatment approaches for OpSCC has propelled research into the molecular landscape of these tumors. Moreover, there is an underrepresentation of genomic data on the mutation profile of HNC patients from admixed populations. Therefore, this study aims at identifying molecular alterations in a set of HNC patients from Brazil and to better characterize, together with clinical variables, OpSCC cases according to their outcome.

## Methods

### Sample population and DNA isolation

This study included pre-treatment formalin-fixed paraffin embedded (FFPE) tissue biopsies from 51 patients with primary oropharyngeal squamous cell carcinoma (OpSCC) diagnosed at the Department of Head and Neck Surgery of Barretos Cancer Hospital in Brazil between 2009 and 2017. These cases were selected based on very stringent criteria to ensure reliability of the clinical and molecular data as follows: pre-treatment FFPE tumor biopsies available from patients treated with curative intent by chemo-radiation protocols and with available follow-up data; samples with at least 60% of tumor cells at the histopathological examination and, with enough DNA for library preparation at an acceptable level of integrity as evaluated by multiplex PCR^25^. Finally, after sequencing, only samples with acceptable scores of DNA sequencing quality were included (as described below).

Hematoxylin & eosin stained sections of paraffin blocks containing the tumor tissue from the patients included were reviewed by two expert pathologists (CSN and GCA) for diagnosis confirmation and characterization of cellular components. Scrapings from regions containing at least 60% of tumor cells were processed using the QIAamp DNA FFPE Tissue Kit (Qiagen, Germany). Isolated DNA samples were eluted in 35 µL of water and quantified in the Qubit fluorometer (Invitrogen, Carlsbad, CA) prior to storage at −20 °C until use.

Information regarding tobacco and alcohol consumption, clinical and pathological features were retrieved from patient’s charts. p16 immunohistochemistry was conducted as a surrogate marker for high-risk HPV (prediluted, monoclonal mouse antihuman p16INK4A protein, Clone E6H4TM, ready for use, Ventana, Tucson, AZ, USA). Samples with strong and diffuse nuclear and cytoplasmic staining in more than 75% of the tumor cells were considered positive for p16^26–28^. Moreover, HPV-DNA detection of types HPV-16 and HPV-18 was performed in a subset of cases (n = 12) using droplet digital PCR as previously described and detailed in the Supplementary Methods^29^.

The present study was approved by the Barretos Cancer Hospital Institutional Review Board (approval number 425/2013) and all methods were performed in accordance with the relevant guidelines and regulations.

### Next-generation sequencing (NGS) and genetic ancestry determination

To identify somatic mutations, we performed NGS of a panel of 15 genes, including some of the most frequently mutated genes in HNC, *TP53, NOTCH1, CDKN2A, PTEN, PIK3CA, FBXW7, HRAS, TP63, CASP8, FAT1, KMT2D, RB1, IRF6, EZH2* and *NSD1*, based on a previous study^30^. Bearing in mind the consensus regarding DNA fragmentation when recovered from FFPE, primers were designed to amplify fragments with sizes ranging from 125–175 base pairs, to minimize the effect of DNA fragmentation in the efficiency of library preparation. Therefore, three pools of primers were used for the simultaneous amplification of 923 amplicons from the 15 genes.

Ten nanograms of genomic DNA from each sample were subjected to library preparation using the Ion AmpliSeq Library Kit 2.0 (Life Technologies, Carlsbad, CA) and an AmpliSeq Custom Panel (Life Technologies, Carlsbad, CA) designed to specifically amplify the entire coding region of the 15 genes, with a coverage of 99.26%. Samples were barcoded using IonXpress Barcode Adapters (Life Technologies, Carlsbad, CA). Automated template preparation and enrichment using an input of 30pM of purified library were performed in an Ion Chef System (Life Technologies, Carlsbad, CA) and sequencing was conducted in an Ion Torrent PGM (Life Technologies, Carlsbad, CA) using the Ion PGM Hi-Q View Sequencing Kit (Life Technologies, Carlsbad, CA).

The genetic ancestry of a subset of patients (n = 11) was determined using AIMs (Ancestry Informative Markers) as previously reported^31–35^. A more detailed description of the methodology used is in the Supplementary Methods.

### Data analysis

Sequencing results obtained in the Ion Torrent PGM run were processed in the Torrent Server. Reads generated were aligned to hg19 human reference genome using the Torrent Mapping Alignment Program (TMAP) and the Torrent Variant Caller (TVC) plugin version v5.8.0.19 (464) was used to call for variants. Variants were annotated using wANNOVAR (http://wannovar.wglab.org/) and Cancer Genome Interpreter^36^. After quality filters were applied per sample (average depth of coverage of at least 600x, at least 80% of reads aligned to the target region and at least 100.000 mapped reads)^37^, variants with the following characteristics were selected: non-synonymous and frameshift variants occurring within exons; variants with a depth of coverage of at least 200x; variants with an allele frequency of at least 10%; frequency observed in population databases of germline variants (such as 1000 genomes, ExAC and ESP6500) lesser than 1%; classified as drivers or predicted drivers using the CGI tool (https://www.cancergenomeinterpreter.org/home); and previously reported at the COSMIC (https://cancer.sanger.ac.uk/cosmic) and/or TCGA databases (http://www.cbioportal.org/).

All remaining variants were manually confirmed on the Integrative Genomics Viewer (IGV) version 2.7. We further explored *TP53* status by using the Evolutionary Action score of *TP53-*coding variants (EAp53) to stratify patients with tumors harboring *TP53* mutations as high or low risk for unfavorable outcome, based on a model of the genotype-phenotype relationship described in^38,39^.

Tobacco, HPV status and mutational data from 39 OpSCC patients evaluated in The Cancer Genome Atlas (TCGA) were accessed with the online Xena Browser (https://xenabrowser.net). Mutation frequencies of the 15 genes tested in this study were compared between both cohorts.

All NGS data generated from the 51 OpSCC samples evaluated here was deposited in the European Genome-phenome Archive (EGA) under study/dataset identifiers EGAS00001004430/EGAD00001006151.

### Statistical analysis

Statistical analysis was performed using the software IBM SPSS Statistics 23 for Windows. Categorical variables were compared using Fisher’s exact or Chi-square test. Survival curves were calculated by Kaplan-Meier method and differences between groups were compared using the log-rank test. Recurrence-free survival was defined as the interval between the date of initial treatment and the diagnosis of recurrence. For all analysis, we considered statistical significance when p value <0.05.

## Results

### Clinical description of the study population

Clinical and histopathological data of the 51 oropharyngeal cancer patients enrolled in this study are presented in Table 1. Most of the patients profiled in this cohort were male (98.0%) with age ranging from 35 to 76 years (mean/median = 55.3/55.0 years). Tobacco and alcohol consumption were self-reported by 86.3% and 96.0% of the cases, respectively. The majority of patients had advanced disease (according to the AJCC TNM 8^th^ edition^40^) at diagnosis (84.3%), T3/T4 tumors (37/51, 72.5%) and with clinically positive lymph nodes at diagnosis (N+; 45/51, 88.2%). All patients were treated with a platin-based chemotherapy concomitant to radiotherapy between 2009 and 2017.

**Table 1:** Clinical and pathological features of oropharyngeal patients (n = 51).

Characteristic	Number of cases	%
**Gender**		
Male	50	98.0
Female	1	2.0
Age		
<=55 years	26	51.0
>55 years	25	49.0
**Smoking status**		
Never	7	13.7
Current or Former	44	86.3
**Alcohol consumption status**		
Never	2	4.0
Current or Former	48	96.0
**HPV status**		
Negative	33	64.7
Positive	18	35.3
**T stage**		
T1/T2	14	27.5
T3/T4	37	72.5
**N stage**		
N0	6	11.8
N+	45	88.2
Stage*		
I/II - early	8	15.7
III/IV – advanced	43	84.3
**Response to treatment**		
Non-responder	19	38.8
Responder	30	61.2
**Recurrence**		
No	37	72.5
Yes	14	27.5
**Live Status**		
Alive without disease	25	49.0
Alive with disease	3	5.9
Death by cancer	23	45.1

*According to the8th edition of the AJCC TNM staging system of oropharyngeal tumorn 40.

The median follow-up was 26 months (range: 1.00 to 86.00 months) and 44.3% of the cases were alive in 5-years. Recurrence occurred in 14 cases (27.5%). Tobacco smoking was significantly associated with reduced recurrence-free survival (50.2% versus 100% for never smokers; log-rank p = 0.045), while HPV-status association with increased recurrence-free survival was marginally significant (75.5% for HPV-positive versus 48.8% for HPV-negative; log-rank p = 0.082) (Supplementary Fig. 1A,B).

The expression of p16 protein was detected in 18/51 samples (35.3%). For a small subset of cases with available DNA (n = 12), droplet digital PCR (ddPCR) was used for the detection of HPV-DNA for types HPV-16 and HPV-18 as part of another study (unpublished data). The agreement between p16-IHC and HPV-DNA results was of 91.7% (Cohen’s Kappa Value = 0.833; p = 0.003): 6 were p16+ /HPV-DNA+, 5 were p16-/HPV-DNA-, but 1 had discordant a result, being p16 + /HPV-DNA- (Supplementary Table 1). Given the high agreement between p16-IHC and HPV-DNA results in the subset of samples tested, and the recommendation of this test as a surrogate marker for HPV-positivity on a clinical setting^41^, we considered patients with tumor p16-IHC expression as positive for HPV infection in this study.

We also included a set of 39 OpSCC with available mutational, tobacco consumption and HPV status (by p16-IHC and/or *in-situ* hybridization) from the TCGA study recovered from the Xena database (Supplementary Table 2). Of the 39 OpSCC cases, 79.5% (31/39) were HPV-positive and 69.2% were current or former smokers (26/39). Therefore, our cohort had a substantially lower rate of HPV-positive cases and higher prevalence of current or former tobacco smoker patients in comparison to this set of patients from the TCGA study.

### Detection of somatic variants and genetic ancestry determination in OpSCC cases

After filtering-out common germline variants, only nonsynonymous variants, with VAF >10%, and previously reported by TCGA in head and neck tumors and/or in COSMIC were considered. The mutation profile identified a total of 136 variants, with an average of 2.67 variants per sample (ranging from 1–31). The majority of variants were classified as missense (117/136; 86.0%), followed by nonsense variants (16/136; 11.8%) and frameshift (3/136; 2.2%). We found an average of 1.65 (ranging from 1–12) genes mutated per sample and the majority of the patients (32/51; 62.7%) were mutated for at least one of the genes tested: 17/32 (53.1%) had only 1 gene mutated, 9/32 (28.1%) had between 2 and 5 genes mutated, and 6/32 (18.8%) had more than 5 genes mutated.

**Figure 1:**
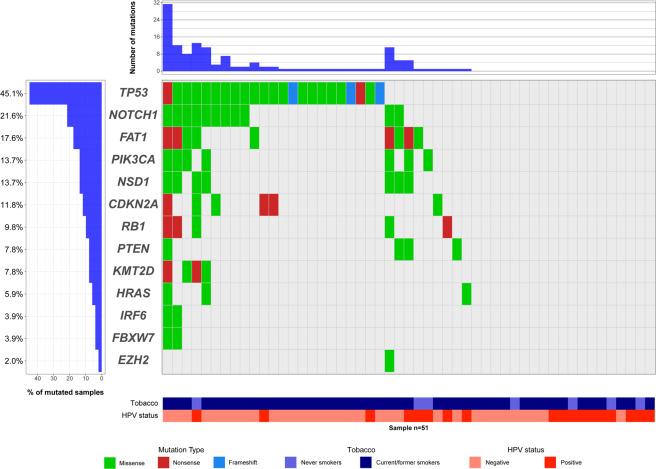
Oncoprint diagram with frequencies and types of mutation observed in OpSCC. Genes (rows) are sorted according to the frequency of mutation within samples (n = 51). Samples (columns) are further classified according to tobacco consumption and HPV status. The right panel represents samples without mutations in the genes tested. Top, number of mutations per sample. Color codes indicate mutation type, tobacco and HPV status. The oncoprint was generated using the package GenVisR (version 1.14) in R 3.5.0 software.

The frequency of mutated samples per gene was as follows: 45.1% (23/51) for *TP53*, 21.6% (11/51) for *NOTCH1*, 17.6% (9/51) for *FAT1*, 13.7% (7/51) for *NSD1* and *PIK3CA*, 11.8% (6/51) for *CDKN2A*, 9.9% (5/51) for *RB1*, 7.8% (4/51) for *KMT2D* and *PTEN*, 5.9% (3/51) for *HRAS*, 3.9% (2/51) for *IRF6 and FBXW7*, and 2.0% (1/51) for *EZH2* (Fig. 1 and Supplementary Fig. 2). None of the patients presented mutations for *CASP8* and *TP63*.

For *TP53*, the most frequently mutated gene in our cohort, most of the mutations were missense (28/51), 5 were truncating (3 frameshift and 2 nonsense) and the majority occurred within the p53 DNA-binding domain (between codons 95 and 288) (Supplementary Fig. 3A). *NOTCH1* was the second most frequently mutated gene in our cohort (11/51) and all variants observed were missense with a high fraction happening in the N-terminal epidermal growth factor (EGF)-like ligand binding domains and another fraction in the C-terminal end, mainly around and within the Ankyrin repeat domain (Supplementary Fig. 3B).

In the set of OpSCC patients from TCGA evaluated, 64.1% of the samples had mutation in at least 1 of the genes tested (25/39), a very similar rate to the one we observed in our cohort (32/51; 62.7%). *TP53* was the most frequently mutated gene (9/39; 23.1%), followed by *PIK3CA* (8/39; 20.5%), *RB1* (5/39; 12.8%), *KMT2D* (4/39; 10.3%), *NOTCH1* and *NSD*1 (3/39; 7.7%), *FAT1, FBXW7* and *PTEN* (2/39; 5.1), and *CDKN2A* (1/39; 2.6%) (Supplementary Fig. 2 and Supplementary Table 2). No mutations in *CASP8*, *EZH2*, *HRAS*, *IRF6* and *TP63* were observed.

Besides somatic variants characterization, for a subset of patients (n = 9) the genetic ancestry was determined (Supplementary Table 1). A great admixture was observed in the genetic composition of the individuals tested based on the four ancestry groups evaluated. As expected, a high proportion of European ancestry was observed (median: 63.8%, range: 42.0–88.5%), followed by African (median: 22.2%, range: 5.5–38.6%), Native American (median: 6.2%, range: 2.0–28.7%) and East Asian (median: 5.0%, range 3.8–23.9%) (Supplementary Table 1 and Supplementary Fig. 4).

### Association between mutation status, HPV status and tobacco consumption

The average number of variants in HPV-negative samples was higher than in HPV-positive cases (3.33 ± 6.04 versus 1.44 ± 3.22; p = 0.199) (Supplementary Fig. 5); and the majority of mutated cases were HPV-negative (24/32, 75.0% versus 8/32, 25.0% for HPV-positive; Chi-square p = 0.046). As expected, genes commonly associated with tobacco-induced HNC carcinogenesis had a lower frequency of mutation in HPV-positive cases (Table 2): 1/18 (5.6%, p = 1.000) for *CDKN2A*, 3/18 (16.7%; p = 1.000) for *FAT1*, 1/18 (5.6%; p = 0.077) for *NOTCH1*, and 3/18 (16.7%; p = 0.003) for *TP53* (Fig. 1 and Supplementary Fig. 6). Interestingly, of the 8 HPV-positive cases harboring mutations, 5 were current or former smokers; and only 2/18 (11.1%) showed mutation in *PIK3CA*, reportedly the most frequently mutated gene in this group (Fig. 1 and Supplementary Fig. 6).

**Table 2:** Results of the association between HPV status and tobacco consumption with the status of somatic gene mutation in the OpSCC patients.

HPV status				Tobacco consumption			
Genes		Negative	Positive	p-value^+^	Never	Current/Former	p-value^+^
*Any gene*	WT	9 (27.3)	10 (55.6)	0.046*	4 (57.1)	15 (34.1)	0.402
	mutated	24 (72.7)	8 (44.4)		3 (42.9)	29 (65.9)	
*CDKN2A*	WT	29 (87.8)	16 (88.9)	1.000	6 (85.7)	39 (88.6)	1.000
	mutated	4 (12.2)	2 (11.1)		1 (14.3)	5 (11.4)	
*FAT1*	WT	27 (81.8)	15 (83.3)	1.000	5 (71.4)	37 (84.1)	0.592
	mutated	6 (18.2)	3 (16.7)		2 (28.6)	7 (15.9)	
*NOTCH1*	WT	23 (69.7)	17 (94.4)	0.072	6 (85.7)	34 (77.3)	1.000
	mutated	10 (30.3)	1 (5.6)		1 (14.3)	10 (22.7)	
*PIK3CA*	WT	28 (84.8)	16 (88.9)	1.000	6 (85.7)	38 (86.4)	1.000
	mutated	5 (15.2)	2 (11.1)		1 (14.3)	6 (13.6)	
*TP53*	WT	13 (39.4)	15 (83.3)	0.003*	6 (85.7)	22 (50.0)	0.112
	mutated	20 (60.6)	3 (16.7)		1 (14.3)	22 (50.0)	

^+^Results obtained using the Fisher´s Exact test, except for* that were obtained with the Chi-square test.

Regarding tobacco consumption, the average number of variants was slightly higher in current or former tobacco smokers when compared with never smokers (2.75 ± 5.38 versus 2.14 ± 4.81; p = 0.780) (Supplementary Fig. 5). Additionally, most of the cases mutated for at least one of the genes tested were current or former smokers (29/32, 90.6% versus 3/32, 9.4% for never smokers; Fisher´s Exact Test p = 0.402). Although not statistically significant, genes commonly associated with tobacco-induced HNC carcinogenesis were more frequently mutated in current or former smokers (Table 2): 10/44 (22.4%; p = 0.592) for *NOTCH1*, and 22/44 (50.0%; p = 0.112) for *TP53* (Fig. 1 and Supplementary Fig. 6). Although the overall frequency of mutations in *CDKN2A* and *FAT1* was slightly higher in never smokers, most of the mutated samples for these genes were current or former smokers: 5/6 (83.3%; p = 1.000) mutated for *CDKN2A* and 7/9 (77.8%; p = 1.000) for *FAT1* (Fig. 1 and Supplementary Fig. 6).

Interestingly, of the 19 cases for which no mutation was detected for the genes tested, all but one patient (18/19; 94.7%) were exposed to at least one of the risk factors evaluated (tobacco smoking and/or positivity for HPV infection): 3 (15.8%) cases were never-smokers, but HPV-positive; 8 were smokers, but HPV-negative; and 7 were smokers and HPV-positive (Fig. 1 and Supplementary Fig. 6).

Additionally, we compared the mutational rates of the 15 genes in our cohort and the 39 OpSCC from the TCGA study. Although the frequency of mutated samples was similar in both cohorts, as described earlier, significant differences in mutation frequencies were observed for specific genes (Supplementary Fig. 2). Genes commonly associated with tobacco-induced HNC carcinogenesis were less frequently mutated in the TCGA samples, namely *TP53* (9/39; 23.1%), *NOTCH1* (3/39; 7.7%), *FAT1* (2/39; 5.1), and *CDKN2A* (1/39; 2.6%); while *PIK3CA* was mutated in 20.5% of the samples (8/39). This agrees with the lower prevalence of ever smokers (69.2% versus 86.3%) and higher prevalence of HPV-positive cases in the TCGA cohort (79.5% versus 35.3%; Supplementary Table 2).

### Association between mutation status and clinical and outcome data

We tested whether the presence of mutation in the genes evaluated could be associated with the following demographical and clinical features: age at diagnosis, gender, tobacco consumption, drinking status, HPV status, T stage, N status and clinical stage. The only statistically significant association found was between *NOTCH1* mutation and tumor size: all tumors with *NOTCH1* mutation (11/11; p = 0.023) had a higher T-stage (T3/T4).

Next, we constructed Kaplan-Meier curves to assess the impact of gene mutations in recurrence-free survival of the patients tested. Although not statistically significant, the presence of mutation in at least one of the genes tested showed a decrease in recurrence-free survival (47.9% versus 80%; log rank p-value = 0.156), the same was observed for the presence of *TP53* (42.6% versus 75.9% for WT; log rank p-value = 0.137) (Table 3).

**Table 3:** Recurrence-free survival information according to selected clinical and molecular factors.

Characteristic	Number of cases	Number of events	5-year recurrence-free survival	p-value (log-rank)
**HPV status**				
Negative	33	11	48.8%	0.082
Positive	18	3	75.5%	
**Tobacco history**				
never	7	0	100.0%	0.045
current/former	44	14	50.2%	
**Mutation status (any gene)**				
WT	19	3	80.0%	0.156
mutated	32	11	47.9%	
***PTEN*** **status**				
WT	47	11	64.5%	0.045
mutated	4	3	25.0%	
***NOTCH1*** **status**				
WT	40	8	68.2%	0.032
mutated	11	6	35.1%	
***TP53*** **status**				
WT	28	5	75.9%	0.137
mutated	23	9	42.6%	
**EAp53**				
WT/low-risk	37	7	71.8%	0.080
high-risk	11	6	38.4%	

**Figure 2:**
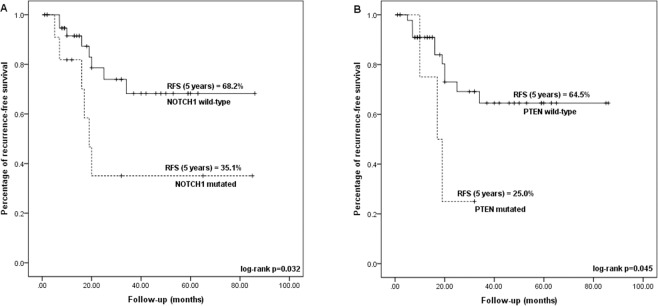
Kaplan Meier curves indicating the difference in 5-year recurrence-free survival (RFS) according to NOTCH1 (**A**) and PTEN (**B**) mutation status. The KM curves were generated using the software IBM SPSS Statistics Version 23.

**Figure 3:**
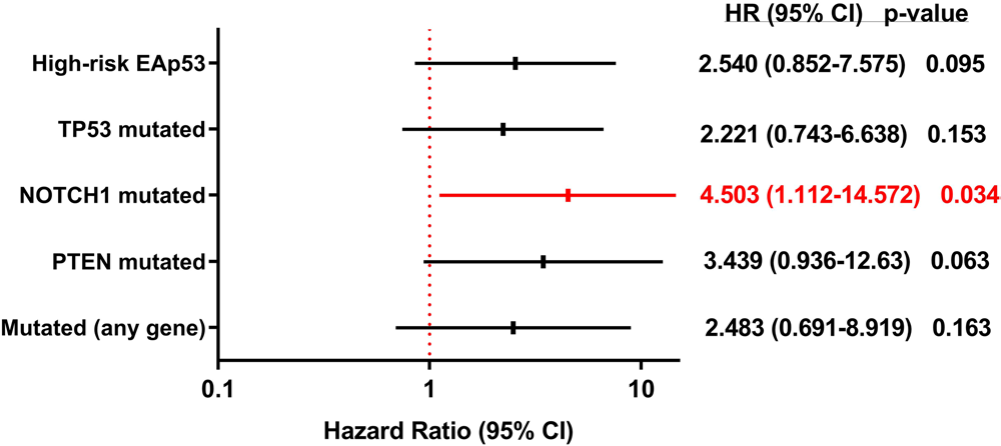
Forest plot with the hazard ratio values and 95% CI for selected molecular alterations (p-values were obtained through Cox regression test). The forest plot was generated using the software IBM SPSS Statistics Version 23.

The presence of mutation in *NOTCH1*, significantly decreased the recurrence-free survival (RFS) of mutated cases (25.1% for mutated cases and 68.2% for WT; log-rank p-test = 0.032) (Table 3 and Fig. 2A). In a multivariate hazard ratio analysis for recurrence, adjusted by HPV and tobacco status, clinical stage, age at diagnosis, *TP53, PTEN* and *NOTCH1* mutation status, only *NOTCH1* mutation remained significant after stepwise selection, with a risk of recurrence of 4.503 for mutated cases (HR 95% CI = 1.112–14.572; Cox Regression p = 0.034) (Fig. 3).

The presence of *PTEN* mutation was also significantly associated with decreased recurrence-free survival (RFS: 25% versus 64.5% in WT; log rank p-test = 0.045) (Table 3 and Fig. 2B). However, the risk estimate by Cox regression was not statistically significant (Fig. 3).

### Breakdown of TP53 effect in clinical outcome

As mentioned earlier, 45.1% (23/51) of the samples tested harbored *TP53* mutations. Although not statistically significant, we observed that the presence of *TP53* variants reduced recurrence-free survival (42.6% versus 75.9% for WT; log rank p-value = 0.137).

We further explored *TP53* missense mutations by using the Evolutionary Action score of *TP53-*coding variants (EAp53)^38,39^ to predict the impact of *TP53* mutations on outcome: 34.8% (8/23) of the mutated cases were scored as having low-risk variants, while 65.2% had high-risk variants. High-risk variants carriers had an increased risk of 2.540-fold of having a recurrence (HR 95% CI = 0.852–7.575; p-value = 0.095) and a marginally significant decreased recurrence-free survival (38.4% versus 71.8%; log rank p-test = 0.080) (Table 3).

## Discussion

Oropharyngeal squamous cell carcinomas arise from epithelial cells of the mucosal lining of the upper aerodigestive tract and, despite all cases developing from one cell type in one tissue, these tumors are remarkably heterogenous. This heterogeneity can be further explained by differences in etiology and in molecular alterations that drive carcinogenesis^42^.

OpSCC were classically associated with heavy tobacco and alcohol consumption; however, a significant decrease in cases related to tobacco/alcohol and an increase in cases related to infection by high-risk human papillomavirus (HPV) is being observed, especially in high and middle-income countries^2,43^. In our cohort, most of the cases were current or former-smokers (86.3%) and only 35.3% were HPV-positive (by p16-IHC); even within the HPV-positive group, the majority of cases were smokers (66.7%). This information shows that tobacco smoking still seems to have a bigger impact in the onset of OpSCC treated at our institution. Despite the small number of cases evaluated in our study, tobacco smoking was significantly associated with reduced recurrence-free survival (RFS), while HPV-status association with increased RFS was marginally significant. It is noteworthy that the majority of HPV-positive cases in our cohort were current or former smokers (12/18; 66.7%). HPV-positive OpSCC has a clearly improved outcome; however, smoking has a reportedly adverse effect on prognosis in both HPV-positive and HPV-negative cases^6^. This may have impacted the survival rates we observed, since although higher than in the HPV-negative group, the difference was not statistically significant.

Brazilian population has been frequently characterized by a considerable admixture of different ancestries in the genetic composition within individuals. Several studies have explored this admixture in large numbers of patients with the most prevalent tumor types (breast^44^, colorectal^31^ and lung^45^), as well as in the healthy population^34^. All these studies found similar results, with a confirmed mixture of ancestry markers for the populations examined and a higher proportion of European ancestry markers, followed by African and then Asian and/or Native American in most of the patients. We were unable to test all our patients for genetic ancestry, but the small subset tested confirmed these results. Previous studies have shown differences in prevalence of mutation in specific genes according to genetic ancestry, such as an enrichment of *TP53* mutations in AFR and of *PIK3CA* in EUR^46^. However, statistically significant confirmation of the genetic ancestry profile of the patients evaluated in this study and testing for any associations with the prevalence of gene mutations would require the evaluation of a substantially larger number of cases.

The most recent genomic progression model for head and neck cancer, resulting from new insights into the cancer genes that are commonly mutated in these tumors, points to different routes towards mucosal squamous cell carcinoma transformation. There seem to be three main genetic subgroups with two of them closely related to the etiology: (i) HPV-related; (ii) tobacco-related; and (iii) HPV-negative/tobacco-negative^42^. Our main findings will be discussed below based on these subgroups.

For HPV-positive cases, transcriptionally-active human papillomavirus leads to cell cycle deregulation through the abrogation of p53 and pRb pathways by the viral oncoproteins E6 and E7 (HPV E6 and E7). Further oncogenic events may lead to differentiation in HPV-KRT (HPV-keratinocyte differentiation and oxidative reduction process) or HPV-IMU (HPV-immune response and mesenchymal cell differentiation) tumors^47^. p16 detection by immunohistochemistry (p16-IHC) was included in the clinical routine as the gold-standard test for HPV-positive oropharyngeal SCC with proved clinical impact^18,48^ due to its easy-of-use, low cost and high sensitivity rates (near 100%)^41,49^. However, it frequently yields not ideal specificity rates (79% and 95%), detecting a positive p16 signal in tumors not associated with HPV^48–50^. For this reason, several studies have suggested additional HPV-specific tests to ensure accurate classification of OpSCC as HPV-related^48,51,52^. In our study, we performed HPV-DNA detection in a subset of the cases and observed a high agreement with p16-IHC results. Moreover, molecular characterization closely agreed with other studies that also used HPV-status to describe results. HPV-positive tumors usually have a lower mutational burden, are usually WT for *TP53* mutations and frequently harbor activating *PIK3CA* mutations and amplifications (mainly the HPV-KRT)^20,22,47,53^. In accordance to this, in our cohort, HPV-negative cases had 2.5-times more variants than HPV-positive cases (3.27 ± 5.854 versus 1.33 ± 3.162, respectively). Moreover, even though most of our HPV-positive cases were smokers (12/18; 66.7%), the frequency of *TP53* mutation in this subgroup was significantly lower (16.7%; p = 0.003). Conversely, we found 2 HPV-positive case with PIK3CA mutation (2/18; 11.1%); a classic C > T mutation in the helical domain, frequent in viral-associated tumors (p.E545K) and commonly associated with the apoliprotein B mRNA-editing enzyme catalytic subunit (APOBEC)-induced mutational signature^24,54^. Hayes and colleagues reported in a review article a frequency between 22–56% of PIK3CA activation in HPV-positive HNC; however, we only evaluated somatic mutations in the present study and did not explore copy-number alterations (CNA). This might explain the lower frequency in comparison to other studies that relate this activation both to mutation and amplification in HPV-positive cases^53^. As expected, besides *TP53*, genes frequently mutated in tobacco-related HNC had a low frequency of mutation in the HPV-positive group, namely *NOTCH1* (1/18; 5.6%), *CDKN2A* (2/18; 11.1%) and *FAT1* (3/18; 16.7%)^53,55^.

The second and most classic subgroup of HNC is associated with tobacco-smoking and also involves deregulation of the cell cycle mainly through loss-of-function (LoF) of two tumor suppressor genes: *TP53* and *CDKN2A*. *CDKN2A* encodes the p16INK4A protein and is lost through deletion, inactivating mutations and hypermethylation in 15–22% of HNC^53^. Together with the frequent amplification of cyclin D1 (*CCND1*), also common in tobacco-associated HNC, *CDKN2A* LoF contributes to unscheduled DNA replication and leads to DNA damage and p53 activation^56^; however, *TP53* inactivation is also found in 60–80% of HNC cases^5,24^, thus abrogating cell cycle arrest and apoptosis^57^. Consequently, these tumors have a higher mutational burden and frequent copy number alterations^42^. In our cohort, we found an overall rate of *CDKN2A* and *TP53* mutation of 9.8% and 45.1%, respectively. Dogan and colleagues found similar rates, when using a targeted exome sequencing approach to characterize 83 HPV-negative and 74 HPV-positive OpSCC^58^: 49% of the cases were mutated for *TP53*, while 22% harbored *CDKN2A* mutations and deletions. Moreover, we did not test for *CDKN2A* inactivation through deletion or hypermethylation. However, among mutated cases, these genes were more frequently mutated in current/former smokers with rates of 83.3% for *CDKN2A* and 95.7% for *TP53*. The breakdown rates according to HPV status were: 78%/16% for TP53 mutations in HPV-negative/positive cases and 39% versus 4% for *CDKN2A* mutations in HPV-negative/positive cases.It is already known that the presence of *TP53* mutations has an important impact in disease progression, treatment response, specially to platinum-based therapy, and survival^59–62^. Although not statistically significant, we observed that the presence of *TP53* variants reduced recurrence-free survival (42.6% versus 75.9% for WT; log rank p-value = 0.137). Next, we explored whether the algorithm Evolutionary Action (EAp53), that stratifies patients according to *TP53* variants associated with especially poor outcomes^38,39^, could be used as a classificatory in our cohort. The association of *TP53* variants classified with EAp53 still did not reach significance, but it is clear that, for our patients, high-risk variants carriers had an increased risk of 2.540-fold of having a recurrence (HR 95% CI = 0.852–7.575; p-value = 0.095) and a marginally significant decreased recurrence-free survival (38.4% versus 71.8%; log rank p-test = 0.080). Previous studies have tested this algorithm in HPV-negative HNC and validated the association of high-risk *TP53* variants, as classified by EAp53, with decreased sensitivity to cisplatin, decreased survival and increased distant metastases in HNC^38,39^. Here we focused our analysis in OpSCC patients with a significant representation of HPV-positive cases and treated by chemoradiation. These differences should be taken into consideration in the interpretation of the algorithm results.

After cell-cycle abrogation, HPV-negative/tobacco-positive cases usually acquire more alterations that drive different routes towards the progression of these tumors. These pathways usually involve squamous differentiation, oxidative stress and WNT signaling. Between 10–21% of HNC demonstrate LoF mutations in *NOTCH1*, impacting squamous differentiation and cell polarity, while 5–23% of cases harbor mutations in *FAT1*, an important component of the WNT signaling^53^. This route seems to impact beta-catenin signaling and keratinocyte transformation, and tobacco-smoking is a known risk factor^42^. We found similar rates of mutated cases for these genes in our cohort, and the majority of cases mutated for *NOTCH1* and *FAT1* were current or former smokers. Moreover, we found and association between *NOTCH1* mutation and a higher T stage, consistent with previous studies showing that functional *NOTCH1* inhibits proliferation in SCC cells and that loss of canonical NOTCH increases tumorigenesis in both HPV-positive and p53-mutant mice^21,63^. In addition, the presence of *NOTCH1* mutations significantly impacted recurrence-free survival. Emerging evidence indicates that Notch effects are dependent on the cellular context in which it is activated, with aberrant Notch signaling being associated with cancer recurrence, metastasis and treatment resistance in different tumor sites^64^. In a recent study, Lim and colleagues tested the feasibility of using targeted NGS to guide treatment of HNC patients, among the genes with association with a poorer overall survival they found *NOTCH1* mutation as a predictor of worse outcome^65^. Similarly, Dong and colleagues found that in HPV-positive OpSCC cases, the presence of *NOTCH1* mutations contributed to a worse overall-survival^58^. Vettore and colleagues also found an association between the presence of mutations in genes of the Notch pathway and a lower disease-free survival in patients with oral tongue carcinoma^66^. In a cohort of esophageal carcinomas from China, Song and colleagues found that patients with mutations in *NOTCH1* had shorter survival and failed to respond to chemotherapy^67^. These results agree with our findings that the status of *NOTCH1* mutations is a promising predictive biomarker for patient outcome and treatment response in OpSCC.

Finally, *PTEN* is a tumor suppressor gene mutated in 9–23% of HPV-negative HNC, leading to oncogenic activity through the activation of the PI3K/AKT/mTOR signaling^42^. We found *PTEN* mutations in 7.8% of the cases tested; once again, this rate does not include CNAs. Interestingly, we observed a significant decrease in recurrence-free survival in the presence of *PTEN* mutation (25% versus 64.5% in WT; log rank p-test = 0.045). A recent study found that patients with high-expressing *PTEN* had an improved progression-free survival in response to cetuximab, in comparison to patients with low expression of this gene^68^. A similar scenario was observed in a Brazilian study on HNC patients treated with cetuximab, with a worse progression-free and overall-survival in patients with loss of *PTEN*^69^.

To our knowledge, this is the first study showing the association between *NOTCH1* and *PTEN* mutations and survival in OpSCC patients treated with platin-based chemotherapy plus radiation.

The third subgroup of HNC comes mainly from previous studies that identified a subset of HNC with a strong correlation between gain of function *HRAS* mutations and LoF mutation in *CASP8*^21,42,70^. This subgroup is usually described as diploid, as having fewer CNAs, DNA mismatch repair proficient and to occur more frequently in women with oral cavity tumors without a history of alcohol and smoking consumption^21,70^. The prevalence rates of this subgroup and the clinical impact is yet to be studied^42^. We did not detect any case with *CASP8* variants in our cohort; moreover, all 3 cases identified with *HRAS* mutation were current or former smokers and one of them was also HPV-positive.

Although differences in the mutation rates of specific genes were observed, mostly due to different prevalence of risk factors (HPV and tobacco smoking), when comparing the results on our cohort and the TCGA OpSCC samples explored, the frequency of cases mutated for at least one of the genes was similar (62.7% versus 64.1%). In the TCGA publication with genomic data on head and neck cancer, only 14% of the samples tested did not present mutation in any gene^24^. Besides including data from all subsites within head and neck cancer, the mutation data reported in the article compiled results from whole exome sequencing, while we only tested a panel of a limited number of genes. This can explain the similarities between mutation rates in the two cohorts of OpSCC samples explored in this article in comparison to the frequency reported in the TCGA publication.

Recently, Perdomo and collaborators, evaluated a similar panel of genes in a cohort comprised of 180 HNC from 3 multicentric studies from South America and Europe^30^. The study reports that 25% of the cases were from the oropharynx (45/180), and only 8% of the entire cohort was HPV-positive (15/180)^30^. Therefore, their cohort was enriched for HPV-negative cases, and, although the number of OpSCC tested was similar to ours, only around 8% were HPV-positive, in contrast to our 35.3% frequency of HPV-positivity. This might explain the slightly lower rates of *TP53* mutations and other genes associated with tobacco-smoking in our cohort in comparison to their findings. In addition, they also explored somatic CNA, that are also included in their higher rates.

Our study has some limitations being the small number of cases evaluated and the short follow-up (median of 26 months) possibly the most important ones, directly impacting the statistical significance of the molecular findings in patient outcome. Besides that, we also did not evaluate copy-number alterations of the genes included, hindering comparisons to other genomic studies that include amplification and deletion in the mutational frequencies. It is important to point out that there is a lack of genomic studies including Brazilian cases; therefore, our study focused in the profile of somatic variants of a set of genes in a cohort of Brazilian OpSCC patients submitted to a homogeneous treatment and with sufficient and well-annotated clinical data. Despite of differences in geographic, economic and social habits when compared to other populations, the results were comparable to those reported before and new insights into possible molecular mechanisms associated with outcome were suggested.

## Conclusions

In conclusion, we report here the results on the genetic characterization of a set of HNC-related genes in a cohort of OpSCC patients. Some of the findings suggest the clinical relevance of genomic approaches to better classify subgroups of patients with different outcomes. Validation of these data could lead to a more refined prognostic stratification and contribute to a personalized treatment approach of OpSCC patients.

### Ethics approval and consent to participate

Informed consent was obtained from each individual prior to sample collection and the study protocol was approved by the Barretos Cancer Hospital Institutional Review Board.

## Supplementary information

Supplementary information.

## Data Availability

All data and materials are available upon request to the Corresponding Author.
