# Supplementary material for: Impact of genetic variants in clinical outcome of a cohort of patients with oropharyngeal squamous cell carcinoma
Source: Sci Rep. 2020 Jun 19;10:9970. doi: 10.1038/s41598-020-66741-z (PMC7305218; doi:10.1038/s41598-020-66741-z)
Supplement: Supplementary file 1 — Supplementary information. [file 41598_2020_66741_MOESM1_ESM.docx]

**Supplementary Info**

**Impact of genetic variants in clinical outcome of a cohort of patients with oropharyngeal squamous cell carcinoma**

Ana Carolina de Carvalho^1*^, Sandra Perdomo^2, 3^, Wellington dos Santos^1^, Gabriela Carvalho Fernandes^4^, Lais Machado de Jesus^1^, Raiany Santos Carvalho^5^, Cristovam Scapulatempo-Neto^1,6^, Gisele Caravina de Almeida^5^, Bruna Pereira Sorroche^1^, Lidia Maria Rebolho Arantes^1^, Matias Eliseo Melendez^1,7,8^, Pedro de Marchi^9,10^, Neil Hayes^11^, Rui Manuel Reis^1,12,13^, André Lopes Carvalho^1,3^

^1^Molecular Oncology Research Center, Barretos Cancer Hospital, Barretos, SP, Brazil; ^2^Institute of Nutrition, Genetics and Metabolism Research, Faculty of Medicine, Universidad El Bosque, Bogotá, Colombia; ^3^International Agency of Research on Cancer, Lyon, France; ^4^Center of Molecular Diagnosis, Barretos Cancer Hospital, Barretos, SP, Brazil; ^5^Research Support Center, Barretos Cancer Hospital, Barretos, SP, Brazil; ^6^Pathology and Molecular Diagnostics Service, Diagnósticos da América-DASA, São Paulo, SP, Brazil; ^7^Pelé Little Prince Research Institute, Curitiba, PR, Brazil, ^8^Little Prince College, Curitiba, PR, Brazil; ^9^Department of Medical Oncology, Barretos Cancer Hospital, Barretos, SP, Brazil; ^10^Oncoclinicas, Rio de Janeiro, RJ, Brazil; ^11^Department of Medicine, Division of Oncology, UTHSC Center for Cancer Research, University of Tennessee Health Science Center, USA; ^12^Life and Health Sciences Research Institute (ICVS), Medical School, University of Minho, Braga; ^13^ICVS/3B's-PT Government Associate Laboratory, Braga/Guimarães, Portugal

**Corresponding author:*

*André Lopes Carvalho, MD, PhD; Molecular Oncology Research Center; Barretos Cancer Hospital*

*Rua Antenor Duarte Vilela, 1331, Barretos, SP 14784-400, Brazil*

*e-mail:* [*carvalhoal@gmail.com*](mailto:carvalhoal@gmail.com)

**Supplementary Methods**

**Genetic Ancestry Determination**

The ancestry of a subset of patients (n=9) was determined using AIMs (Ancestry Informative Markers) as previously reported [^32-36^](#_ENREF_32). Briefly, 46 small INDEL (Insertion-Deletion) polymorphisms, informative for admixed populations, were used to estimate ancestry proportions of four human major population groups: Native American (AME), European (EUR), African (AFR) and East Asian (ASN) and carefully. After multiplex PCR amplification, products were subjected to capillary electrophoresis and fragment analysis on an ABI 3500 Genetic Analyzer (Applied Biosystems) according to the manufacturer's instructions. The ancestry ratios were evaluated using the Structure Software v2.3.4 [^33^](#_ENREF_33)^,^[^34^](#_ENREF_34)^,^[^37^](#_ENREF_37)^,^[^38^](#_ENREF_38), considering the four main population groups (AME, EUR, AFR and ASN), as possible contributors to the current Brazilian genetic composition.

**HPV-DNA testing by droplet digital PCR**

HPV-DNA detection of types HPV-16 and HPV-18 was performed in a subset of cases (n=12) using droplet digital PCR (ddPCR) as previously described. [^31^](#_ENREF_31) Briefly, PCR reactions were performed with 2X ddPCR Supermix for Probes (No dUTP) (BioRad, USA), 900nM of each primer and 300 nM of probe in a total volume of 22µL followed by droplet generation using an automated droplet generator (BioRad, USA). Cycling conditions were as follows: enzyme activation at 95˚C for 10 minutes followed by 40 cycles of denaturation at 94˚C for 30 seconds, annealing/extension for 60 seconds at 58˚C and final enzyme deactivation at 98˚C for 10 minutes. After amplification, PCR plates were transferred to a QX200 droplet reader (BioRad, USA), and fluorescence amplitude and the number of copies of HPV DNA was obtained by QuantaSoft software (BioRad, USA).


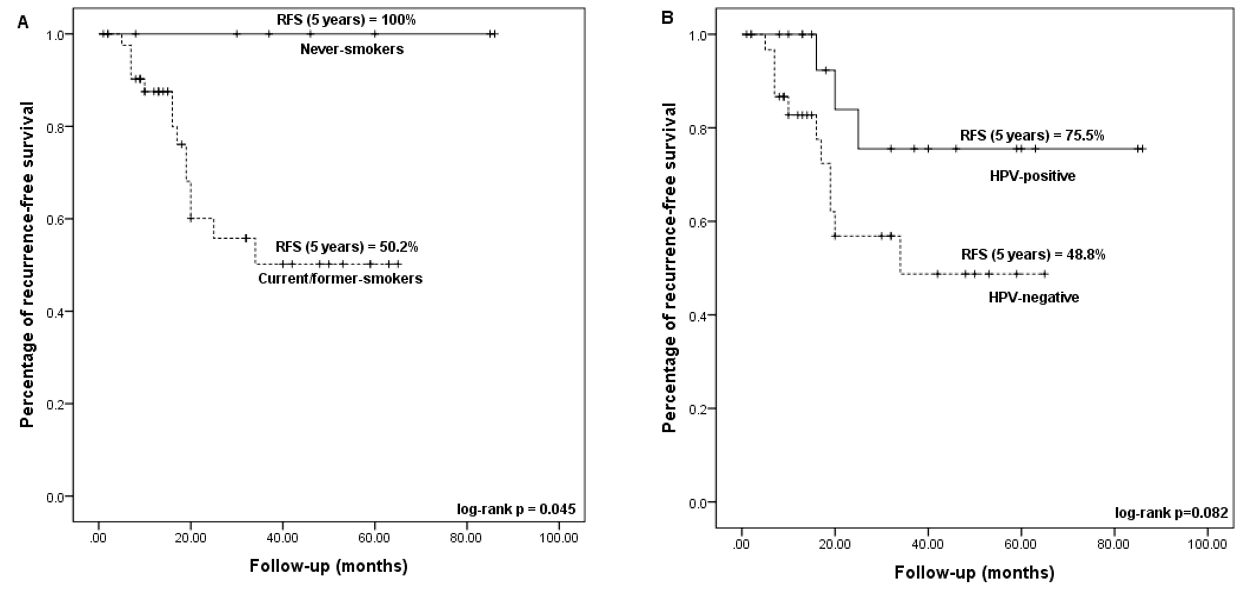


***Supplementary Figure 1.*** *Kaplan Meier curves indicating the difference in 5-year recurrence-free survival (RFS) according to tobacco consumption (A) and HPV status (B). KM curves were generated using the software IBM SPSS Statistics Version 23.*

*
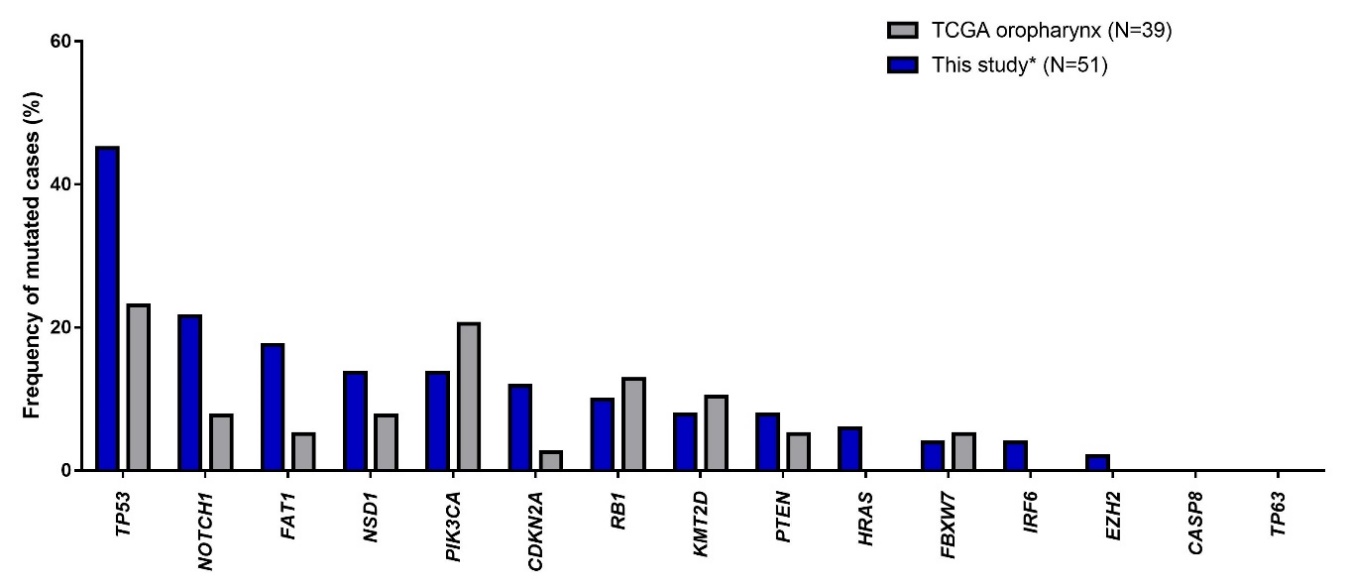
*

***Supplementary Figure 2.*** *Frequency of mutated cases in the 15 genes tested the 51 OpSCC evaluated in this study (blue bars) and 39 OpSCC evaluated in TCGA study (grey bars). The graph was generated using the software GraphPad Prism 7 for Windows.*


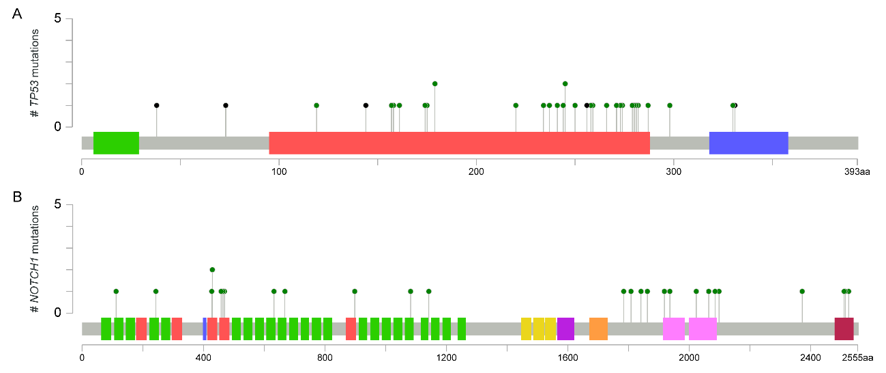


***Supplementary Figure 3.*** *Mutation mapper with the distribution of the variants according to different domains of the two most frequently mutated genes in our cohort: TP53 (A) and NOTCH1 (B). Each bar represents a different protein domain. Each lollipop represents the position of a variant found: the height represents the number of mutations observed and the color represents the type of predicted coding impact of the variant (green circles: missense mutations; black circles: truncating mutations, either nonsense or frameshift).* *Mutation mappers were generated using the corresponding tool at cBioPortal* (<http://www.cbioportal.org/>)*.*

***
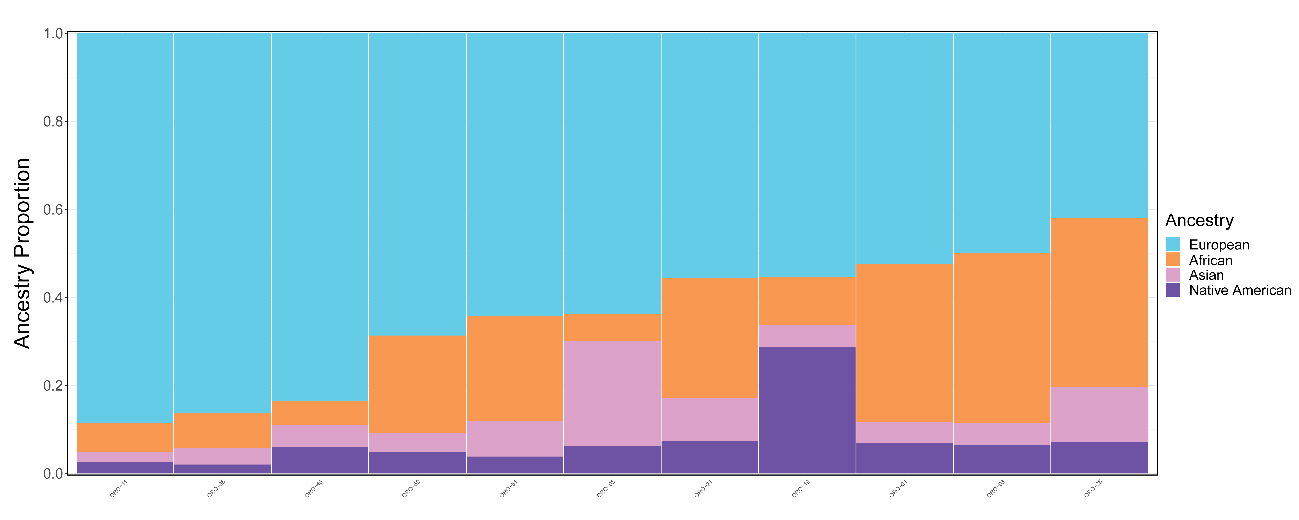
Supplementary Figure 4.*** *Representation of individual ancestry proportions of Brazilian OpSCC patients (n=9) based on the European (blue), African (orange), Asian (pink) and Native American (blue) ancestry populations. The* *ancestry chart was generated using ggplot2 package (version 3.1.0) in R 3.5.0 software.*


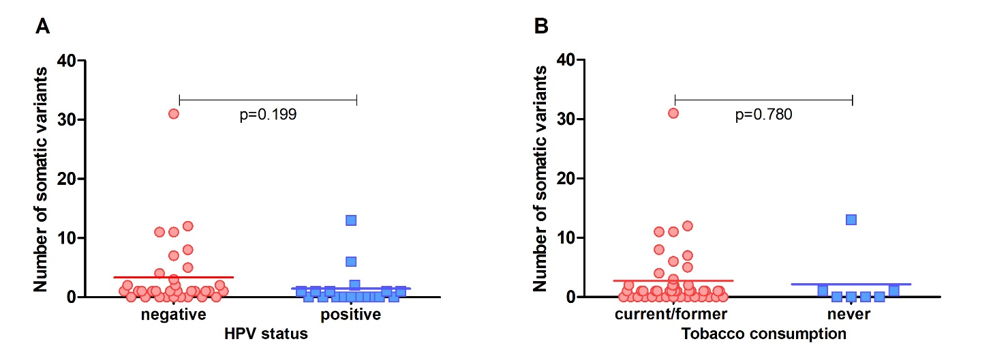


***Supplementary Figure 5.*** *Number of somatic variants identified in the samples tested according to HPV status (A) and tobacco consumption (B). These images were generated using the software GraphPad Prism 7 for Windows.*

*
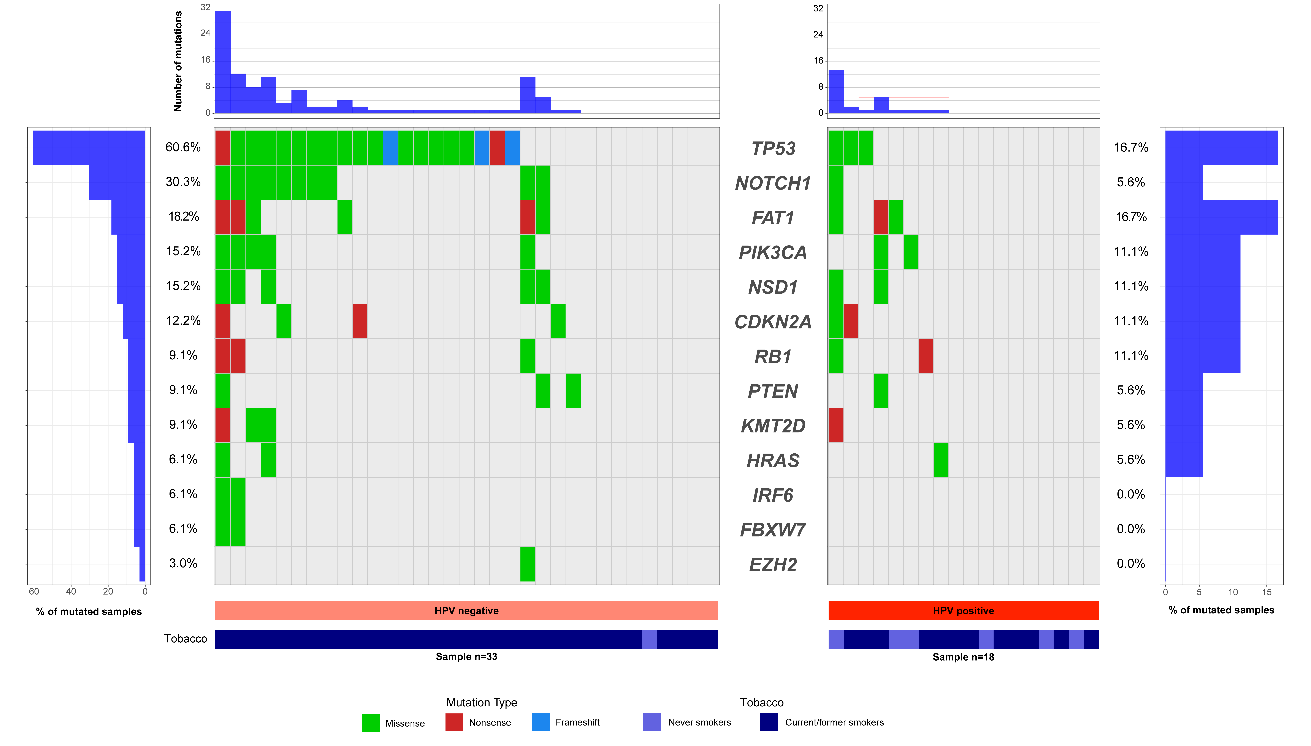
*

***Supplementary Figure 6.*** *Oncoprint diagram with frequencies and types of mutation observed in OpSCC. Genes (rows) are sorted according to the frequency of mutation within samples (n=51). Samples (columns) are further classified according to tobacco consumption and HPV status. The right panel represents samples without mutations in the genes tested. Top, number of mutations per sample. Color codes indicate mutation type, tobacco and HPV status. Image was generated using the package GenVisR (version 1.14) in R 3.5.0 software.*

***Supplementary Table 1.*** *Description of findings of the detection of p16 expression by IHC, HPV-DNA by droplet digital PCR and the proportions of African (AFR), European (EUR), East Asian (ASN) and Native American (AME) ancestries for each OpSCC cases tested.*

| **Molecular ID** | **p16-IHC** | **HPV-DNA** | **AFR** | **EUR** | **ASN** | **AME** |
| --- | --- | --- | --- | --- | --- | --- |
| **ORO-03** | pos | HPV-16 | 0.386 | 0.499 | 0.05 | 0.065 |
| **ORO-25** | pos | HPV-16 | 0.383 | 0.42 | 0.126 | 0.071 |
| **ORO-19** | pos | HPV-16 | 0.109 | 0.554 | 0.05 | 0.287 |
| **ORO-11** | pos | HPV-16 | 0.066 | 0.885 | 0.023 | 0.026 |
| **ORO-50** | pos | neg | 0.222 | 0.686 | 0.042 | 0.049 |
| **ORO-45** | pos | n/a | 0.055 | 0.835 | 0.05 | 0.06 |
| **ORO-01** | neg | neg | 0.358 | 0.525 | 0.048 | 0.069 |
| **ORO-21** | neg | neg | 0.272 | 0.557 | 0.098 | 0.074 |
| **ORO-36** | neg | neg | 0.079 | 0.862 | 0.038 | 0.02 |
| **ORO-05** | neg | neg | 0.061 | 0.638 | 0.239 | 0.062 |
| **ORO-51** | neg | n/a | 0.239 | 0.642 | 0.081 | 0.038 |
| **ORO-39** | pos | HPV-16 | n/a | n/a | n/a | n/a |
| **ORO-13** | pos | HPV-16 | n/a | n/a | n/a | n/a |
| **ORO-09** | neg | neg | n/a | n/a | n/a | n/a |

*Legend: pos, positive for p16-IHC; neg, negative for p16-IHC or HPV-DNA; HPV-16, positive for HPV-16 DNA; n/a, not available*

***Supplementary Table 2.*** *Detailed information of the tobacco consumption history, HPV status, HNC subsite and mutation status (mut=mutated, wt=wild type) for the 15 genes of interest in the current study of the 38 OpSCC patients from the TCGA evaluated. Data were retrieved from the Xena browser.*

| Sample ID TCGA | Tobacco history | HPV status | ICD | *TP53* | *KMT2D* | *FAT1* | *CDKN2A* | *NOTCH1* | *PTEN* | *PIK3CA* | *FBXW7* | *HRAS* | *TP63* | *CASP8* | *RB1* | *IRF6* | *EZH2* | *NSD1* |
| --- | --- | --- | --- | --- | --- | --- | --- | --- | --- | --- | --- | --- | --- | --- | --- | --- | --- | --- |
| TCGA-BA-A4IF-01 | former | Negative | C10.3 | mut | wt | mut | mut | mut | wt | mut | wt | wt | wt | wt | wt | wt | wt | mut |
| TCGA-BA-A4IH-01 | former | Positive | C10.9 | wt | wt | wt | wt | wt | wt | wt | wt | wt | wt | wt | wt | wt | wt | wt |
| TCGA-BA-A6DL-01 | current | Negative | C10.9 | mut | wt | wt | wt | wt | wt | wt | wt | wt | wt | wt | wt | wt | wt | mut |
| TCGA-BA-A8YP-01 | current | Negative | C10.9 | mut | wt | wt | wt | wt | wt | mut | wt | wt | wt | wt | wt | wt | wt | wt |
| TCGA-BB-4228-01 | current | Positive | C01.9 | wt | wt | wt | wt | wt | wt | wt | mut | wt | wt | wt | wt | wt | wt | wt |
| TCGA-BB-A6UM-01 | former | Positive | C09.9 | wt | wt | wt | wt | wt | mut | wt | wt | wt | wt | wt | wt | wt | wt | wt |
| TCGA-CN-5374-01 | current | Positive | C09.9 | wt | wt | wt | wt | wt | wt | wt | wt | wt | wt | wt | wt | wt | wt | wt |
| TCGA-CN-A499-01 | never | Positive | C09.9 | wt | wt | wt | wt | wt | wt | wt | wt | wt | wt | wt | mut | wt | wt | wt |
| TCGA-CN-A49C-01 | former | Positive | C09.9 | wt | wt | wt | wt | wt | wt | wt | wt | wt | wt | wt | wt | wt | wt | wt |
| TCGA-CN-A63Y-01 | never | Positive | C09.9 | wt | wt | wt | wt | wt | wt | wt | wt | wt | wt | wt | wt | wt | wt | wt |
| TCGA-CN-A6UY-01 | former | Positive | C01.9 | wt | wt | wt | wt | wt | wt | wt | wt | wt | wt | wt | wt | wt | wt | wt |
| TCGA-CN-A6V1-01 | never | Positive | C09.9 | wt | wt | wt | wt | wt | wt | mut | wt | wt | wt | wt | wt | wt | wt | wt |
| TCGA-CN-A6V6-01 | former | Positive | C01.9 | wt | mut | wt | wt | wt | wt | wt | wt | wt | wt | wt | wt | wt | wt | wt |
| TCGA-CN-A6V7-01 | never | Positive | C09.9 | wt | wt | wt | wt | wt | wt | wt | mut | wt | wt | wt | wt | wt | wt | wt |
| TCGA-CR-5250-01 | current | Positive | C01.9 | wt | wt | wt | wt | wt | wt | wt | wt | wt | wt | wt | wt | wt | wt | wt |
| TCGA-CR-6477-01 | former | Negative | C01.9 | mut | wt | mut | wt | wt | wt | wt | wt | wt | wt | wt | wt | wt | wt | wt |
| TCGA-CR-6480-01 | never | Positive | C09.9 | wt | wt | wt | wt | wt | wt | wt | wt | wt | wt | wt | wt | wt | wt | wt |
| TCGA-CR-6481-01 | never | Positive | C09.9 | wt | wt | wt | wt | mut | wt | wt | wt | wt | wt | wt | wt | wt | wt | mut |
| TCGA-CR-6482-01 | former | Positive | C09.9 | wt | wt | wt | wt | wt | wt | wt | wt | wt | wt | wt | wt | wt | wt | wt |
| TCGA-CR-6487-01 | former | Positive | C09.9 | wt | mut | wt | wt | wt | wt | mut | wt | wt | wt | wt | mut | wt | wt | wt |
| TCGA-CR-7404-01 | never | Positive | C09.9 | wt | wt | wt | wt | wt | wt | mut | wt | wt | wt | wt | wt | wt | wt | wt |
| TCGA-DQ-7591-01 | former | Positive | C01.9 | wt | wt | wt | wt | wt | wt | wt | wt | wt | wt | wt | wt | wt | wt | wt |
| TCGA-DQ-7593-01 | current | Positive | C01.9 | wt | mut | wt | wt | wt | wt | wt | wt | wt | wt | wt | wt | wt | wt | wt |
| TCGA-F7-A61V-01 | current | Negative | C01.9 | mut | wt | wt | wt | wt | wt | wt | wt | wt | wt | wt | wt | wt | wt | wt |
| TCGA-H7-A76A-01 | former | Positive | C09.9 | wt | wt | wt | wt | wt | wt | wt | wt | wt | wt | wt | wt | wt | wt | wt |
| TCGA-HD-8224-01 | never | Negative | C01.9 | mut | wt | wt | wt | wt | wt | wt | wt | wt | wt | wt | wt | wt | wt | wt |
| TCGA-HD-8314-01 | never | Positive | C01.9 | wt | wt | wt | wt | wt | wt | wt | wt | wt | wt | wt | wt | wt | wt | wt |
| TCGA-HD-A634-01 | current | Positive | C09.9 | wt | wt | wt | wt | wt | wt | wt | wt | wt | wt | wt | wt | wt | wt | wt |
| TCGA-KU-A6H7-01 | former | Positive | C09.9 | wt | wt | wt | wt | wt | wt | wt | wt | wt | wt | wt | mut | wt | wt | wt |
| TCGA-MZ-A5BI-01 | unknown | Positive | C09.9 | wt | wt | wt | wt | wt | wt | wt | wt | wt | wt | wt | wt | wt | wt | wt |
| TCGA-MZ-A6I9-01 | former | Positive | C10.9 | wt | wt | wt | wt | wt | wt | mut | wt | wt | wt | wt | wt | wt | wt | wt |
| TCGA-MZ-A7D7-01 | current | Negative | C01.9 | mut | wt | wt | wt | wt | wt | wt | wt | wt | wt | wt | wt | wt | wt | wt |
| TCGA-QK-A6IF-01 | never | Positive | C09.9 | wt | wt | wt | wt | wt | wt | wt | wt | wt | wt | wt | wt | wt | wt | wt |
| TCGA-QK-A6V9-01 | never | Positive | C10.9 | wt | wt | wt | wt | wt | wt | wt | wt | wt | wt | wt | mut | wt | wt | wt |
| TCGA-QK-A8ZA-01 | current | Positive | C10.9 | mut | wt | wt | wt | mut | wt | wt | wt | wt | wt | wt | wt | wt | wt | wt |
| TCGA-RS-A6TP-01 | former | Positive | C09.9 | wt | mut | wt | wt | wt | wt | wt | wt | wt | wt | wt | wt | wt | wt | wt |
| TCGA-T2-A6WZ-01 | current | Negative | C01.9 | mut | wt | wt | wt | wt | wt | mut | wt | wt | wt | wt | mut | wt | wt | wt |
| TCGA-T2-A6X0-01 | former | Positive | C09.9 | wt | wt | wt | wt | wt | mut | wt | wt | wt | wt | wt | wt | wt | wt | wt |
| TCGA-TN-A7HI-01 | current | Positive | C09.9 | wt | wt | wt | wt | wt | wt | mut | wt | wt | wt | wt | wt | wt | wt | wt |
